# Dietary intake of creatine and risk of medical conditions in U.S. older men and women: Data from the 2017–2018 National Health and Nutrition Examination Survey

**DOI:** 10.1002/fsn3.2543

**Published:** 2021-08-25

**Authors:** Sergej M. Ostojic, Darinka Korovljev, Valdemar Stajer

**Affiliations:** ^1^ FSPE Applied Bioenergetics Lab University of Novi Sad Novi Sad Serbia

**Keywords:** angina pectoris, creatine, elderly, malnutrition, National Health and Nutrition Examination Survey

## Abstract

We examined dietary intake of creatine in U.S. men and women aged 65 years and over, and evaluated the association between creatine intake and risk of self‐reported medical conditions, and physical functioning/disability variables using data from the 2017–2018 National Health and Nutrition Examination Survey (NHANES). The NHANES 2017–2018 target population included the noninstitutionalized civilian resident population of the United States aged 65 years and over. Detailed dietary intake data from NHANES elderly were obtained by dietary interview component through a 24‐h dietary recall interview, with estimated individual values for total grams of creatine consumed per day for each respondent. A threshold for dietary intake of creatine used to calculate risk between creatine intake and medical conditions was set at 1.00 g/day. The sample population included 1500 participants aged 65 years and older, of which 1221 individuals (627 men and 594 women) provided detailed dietary data via a dietary interview. Creatine intake across all participants was 0.76 ± 0.79 g/day (95% CI from 0.72 to 0.81). As much as 70% of U.S. elderly consume <1.00 g of creatine per day, with about 1 in 5 individuals (19.8%) consume no creatine at all. Elderly with the suboptimal intake of creatine were found to have 2.62 times higher risk of angina pectoris (adjusted OR = 2.62, 95% CI from 1.14 to 6.01, *p* = .023) and 2.59 times higher risk of liver conditions (adjusted OR = 2.59, 95% CI from 1.23 to 5.48, *p* = .013), compared with older counterparts who consume ≥1.00 g of creatine per day after controlling for demographic and nutritional variables. The considerable shortage of dietary creatine is associated with an increased risk of heart and liver conditions, which calls for public measures that foster diets rich in creatine‐containing foods, and additional research to investigate the role of creatine in age‐related diseases.

## Graphical abstract

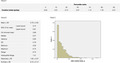

## 1 INTRODUCTION

Elderly individuals are at risk for malnutrition due to a plethora of medical, psychological, lifestyle, and social factors (Hickson, 2006). Malnutrition can aggravate many age‐related medical conditions, inflate the burden of noncommunicable chronic diseases, and compromise the health‐related quality of life in this sensible population (Newberry & Dakin, 2021). This calls for rather opportune measures to identify, monitor, manage, and prevent any nutritional deficiency in the elderly. Among other types of malnutrition, older adults (defined as age >65 years) often ingest less than recommended daily allowances for protein and proteinogenic amino acids (Bauer et al., 2013). A shortfall of food protein supplies relative to needs leads to low protein turnover, muscle wasting, and other protein–malnutrition traits (Corish & Bardon, 2019). However, nonproteinogenic amino acids are also common in human foods and can play significant roles in human metabolism during aging (Dato et al., 2019). Whether nonproteinogenic amino acids are adequately supplied by a regular diet in seniors remains poorly addressed so far, along with unknown risk(s) of inadequate provision for elderly health and everyday life.

Creatine (α‐methyl‐guanidinoacetic acid) is an endogenous nonproteinogenic amino acid derivative that is also found in foods rich in animal protein, including fish, red meat, and poultry. Being a conditionally essential nutrient, creatine plays a key role in energy metabolism as a temporal/spatial energy buffer and metabolic regulator (Wallimann et al., 2011). The daily turnover of creatine is ~2 g of which one half is produced inside the human body from glycine, arginine, and methionine, and another half (about 1 g) comes from an omnivorous diet (Wyss & Kaddurah‐Daouk, 2000). That being said, low‐creatine availability could be triggered either by defective endogenous production or by limited dietary provision of creatine, which provokes various disorders that primarily affect energy‐demanding tissues (Blancquaert et al., 2018; Schulze, 2013). Therefore, eating a low‐creatine diet might contribute to creatine malnutrition in elderly. On the other hand, the elderly could benefit from creatine consumption to combat age‐associated changes in body composition and functional performance (Antonio et al., 2021; Chami & Candow, 2019; Dolan et al., 2019; Forbes et al., 2021; Gualano et al., 2016), suggesting a critical role of consuming this nonproteinogenic compound in older adults. Nevertheless, the population‐based studies omitted to discern an average dietary intake of creatine in older people, and perhaps quantify the extent of suboptimal creatine intake and further assess the possible link between average daily creatine intake and medical conditions in the elderly. This study examines dietary intake of creatine in U.S. men and women aged 65 years and over, and evaluates the connection between creatine consumption and risk of self‐reported medical conditions, using data from the 2017–2018 National Health and Nutrition Examination Survey (NHANES).

## 2 METHODS

### 2.1 Study population

Data for this study were obtained from the NHANES 2017–2018 round. The NHANES is an annual survey research program conducted by the National Center for Health Statistics (NCHS), a public agency of the U.S. Federal Statistical System, to assess and monitor the health and nutritional status of adults and children in the United States. The survey combines interviews, physical examinations, and laboratory tests conducted by trained health professionals at home and in mobile examination centers. The NHANES target population comprises the noninstitutionalized civilian resident population of the United States, with the NHANES 2017–2018 sample included a total of 9245 male and female respondents aged 0–80 years. The sample for NHANES was selected using a complex, four‐stage sample design, with sample weights were used to produce estimates of the health‐related statistics that would have been obtained whether the entire sampling frame had been surveyed (Chen et al., 2020). For this analysis, we singled out data for adult respondents aged 65 years and over at the time of screening. The ethical approval to conduct the current round of NHANES 2017–2018 was granted by the U.S. NCHS Research Ethics Review Board (#2018‐01 and #2011‐17), and informed consent was obtained from all respondents.

### 2.2 Dietary data and creatine intake

Detailed dietary intake data from NHANES 2017–2018 elderly cohort was obtained by a dietary interview component through a 24‐h dietary recall interview. Individual data files that contained detailed information about each food item consumed (including the description, amount of, and nutrient content) reported by each participant were extracted and summarized to estimate the personal intakes of energy, macronutrients, and macronutrient components from those foods and beverages. The U.S. Department of Agriculture (USDA) Food Surveys Research Group was responsible for the dietary data collection methodology, maintenance of the databases used to code and process the data, and data review and processing, with detailed information available elsewhere (NCHS, 2020). To calculate creatine intake, we first identified meat‐based protein foods using 8‐digit USDA food codes organized in five database subgroups (e.g., meat, organ meats, frankfurters, sausage and luncheon meats, poultry, fish, and shellfish) using dietary interview entries for individual foods. We subsequently recorded the gram weight of each food component containing meat‐based protein and calculated the net intake of meat‐based protein for each respondent by merging all relevant food items on daily basis. Individual values for total grams of creatine consumed per day for each respondent were computed using the average amount of creatine (3.88 g/kg) across all sources of meat‐based protein, as previously described (Bakian et al., 2020). Afterward, all participants were separated into four categories of creatine intake, including no‐intake group (0.00 g/day), low‐intake group (0.01–0.99 g/day), medium‐intake group (1.00–1.99 g/day), and high‐intake group (≥2 g/day), with detailed demographic and nutritional profiles described for each category. This stratification was chosen as biologically plausible due to the fact that literature advices for 1–2 g of dietary creatine as daily needs for the general population (Brosnan et al., 2011). In addition, a threshold for dietary intake of creatine used to calculate risk between creatine intake and health conditions (see below) was set at 1.00 g/day, with respondents were classified into two separate subpopulations with the suboptimal intake of creatine (<1.00 g/day) or recommended intake (dietary creatine ≥1.00 g/day).

### 2.3 Questionnaire data

Data collected from NHANES 2017–2018 health conditions and physical disabilities questionnaires were acquired for two subpopulations with calculated suboptimal or recommended dietary creatine intake. Potentially relevant variables from Medical Conditions (MCQ), Cardiovascular Disease and Health (CDQ), Diabetes (DIQ), Osteoporosis (OSQ), Disability (DLQ), and Physical Functioning (PFQ) were identified for further analysis. The MCQ section provided self‐ and proxy‐reported personal interview data on a broad range of health conditions and medical history for NHANES participants, with the section, generally modeled on the medical condition questionnaire section of the U.S. National Health Interview Survey (Miller et al., 2013). The CDQ section provided participant‐level interview data on evaluating cardiovascular health and included questions to assess the presence of angina pectoris as defined by the Rose Angina Questionnaire (Rose, 1962). The DIQ section provided personal interview data on diabetes, prediabetes, and other related issues associated with diabetes. The OSQ section provided personal interview data on several issues related to osteoporosis and brittle bones, with relevant variables used in this report include self‐reported information regarding fractured hip, wrist, or spine, and whether the participant has ever been diagnosed as having osteoporosis. The DLQ questionnaire provides respondent‐level interview data on serious difficulty hearing, seeing, concentrating, walking, dressing, and running errands. The PFQ section provides respondent‐level interview data on functional limitations caused by long‐term physical, mental, and emotional problems or illnesses. All questions were asked in the home by trained interviewers using the computer‐assisted personal interview (CAPI) system, and binary threshold codes (YES/NO) were selected to define the presence of each relevant condition in this cohort, while other codes (e.g., refused, do not know, and missing) were excluded from further analysis. The data were additionally reviewed for completeness, consistency, and illogical values. Further details of the questionnaire instruments and data protocol are documented elsewhere (CDC/National Center for Health Statistics, 2020).

### 2.4 Statistical analyses

National Health and Nutrition Examination Survey complex sampling design was used for data management and analyses. A descriptive analysis comparing demographic and nutritional variables across four groups of dietary creatine intake was conducted using adjusted chi‐square tests or Fisher's exact tests. Single and multivariable logistic regression analyses were conducted to assess the association between subsamples with suboptimal and recommended dietary creatine intake and health conditions. The regression models were adjusted for an a priori defined set of covariates, including age, gender, body mass index, energy intake, and total protein. Data were analyzed using SPSS Statistics for Mac (Version 24.0; IBM), with the significance level set at *p* < .05.

## 3 RESULTS

**FIGURE 1:**
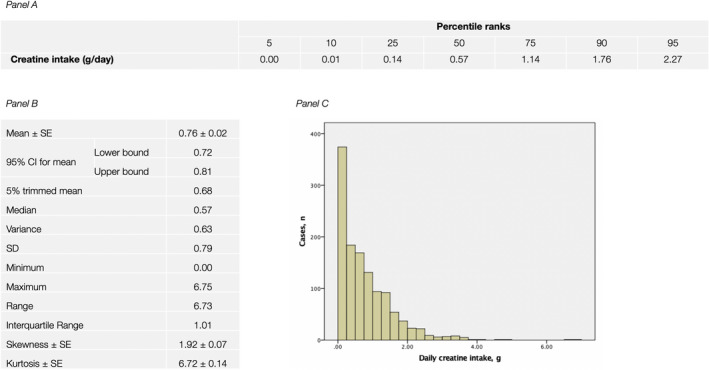
The percentile ranks (Panel a), descriptive statistics (Panel b), and histogram (Panel c) for daily dietary intake of creatine across NHANES 2017–2018 men and women aged 65 years and over (*n* = 1221). CI, confidence interval; SD, standard deviation; SE, standard error

The NHANES 2017–2018 population included 1500 participants aged 65 years and older, of which 1221 individuals (627 men and 594 women; age 73.2 ± 5.3 years; body mass index 29.4 ± 6.5 kg/m^2^) provided detailed dietary data via a dietary interview. A total of 979 respondents (80.2%) took at least 0.02 g of creatine daily from food sources, while 242 respondents (19.8%) consumed no dietary creatine. Average creatine intake across all participants was 0.76 ± 0.79 g/day (95% confidence interval [CI] from 0.72 to 0.81) and 10.2 ± 11.4 mg/kg body weight (95% CI from 9.6 to 10.8). The percentile ranks with weighted averages, descriptive statistics, and histogram for daily dietary intake of creatine in NHANES 2017–2018 elderly cohort were depicted in Figure 1.

The general and dietary characteristics of study participants for a total sample and across four different categories of daily creatine intake were depicted in Table 1. Cohort profiles were similar across categories for most characteristics (*p* > .05); however, the distribution of participants in various categories of creatine intake was significantly different (*p* < .001). Specifically, the participants who consumed no creatine at all combined with those who consumed <1 g of creatine per day significantly outnumbered participants from the other two categories merged (70.0% vs. 30.0%; *p* < .001). The differences were also found for almost all dietary characteristics (except for total sugars), with values for daily energy intake and macronutrients successively higher across creatine intake categories from no‐creatine participants to high‐intake creatine subsample (*p* < .001).

**TABLE 1:** General and dietary characteristics of creatine intake in elderly aged 65 years and above^a^

Characteristics	Total sample	No intake	Low intake	Medium intake	High intake	p*
Cohort profile						
Participants, *n*	1221	242	613	279	87	<.001
Females, %	48.6	50.0	47.0	49.1	55.2	.500
Age, years	73.2 ± 5.3	73.2 ± 5.4	73.1 ± 5.3	73.3 ± 5.3	73.3 ± 5.4	.981
Weight, kg	79.9 ± 20.1	81.6 ± 23.0	79.6 ± 19.1	79.4 ± 19.9	78.3 ± 19.4	.627
Height, cm	164.5 ± 9.9	164.9 ± 10.1	164.6 ± 9.7	164.4 ± 10.0	163.1 ± 10.2	.395
BMI, kg/m^2^	29.4 ± 6.5	29.8 ± 7.3	29.3 ± 6.4	29.3 ± 6.4	29.2 ± 5.4	.850
WC, cm	103.5 ± 14.7	104.5 ± 15.2	103.2 ± 14.2	103.1 ± 15.5	103.9 ± 14.8	.699
Overweight, %	74.4	78.6	76.5	72.7	78.2	.425
Daily dietary intake						
Creatine, g	0.76 ± 0.79	0.00 ± 0.00	0.50 ± 0.26	1.40 ± 0.27	2.76 ± 0.86	<.001
Energy intake, kcal	1877 ± 859	1458 ± 716	1775 ± 773	2212 ± 861	2696 ± 883	<.001
Total protein, g	69.9 ± 38.8	38.4 ± 22.9	59.8 ± 25.7	95.3 ± 25.8	147.3 ± 37.5	<.001
Meat protein, g	37.0 ± 30.2	0.00 ± 0.00	19.2 ± 10.0	54.1 ± 10.3	107.2 ± 33.4	<.001
Total carbohydrates, g	222.4 ± 110.1	204.2 ± 99.0	217.6 ± 106.0	240.5 ± 124.3	248.6 ± 108.2	<.001
Total sugars, g	95.1 ± 62.7	93.9 ± 57.3	92.9 ± 62.1	99.4 ± 69.5	99.7 ± 58.1	.341
Dietary fiber, g	16.1 ± 10.1	15.2 ± 9.8	15.6 ± 10.0	17.3 ± 10.1	18.1 ± 10.5	.002
Total fat, g	77.7 ± 42.6	54.8 ± 34.4	72.8 ± 37.9	94.9 ± 40.8	119.9 ± 49.1	<.001
Saturated fats, g	24.9 ± 15.3	17.9 ± 12.9	23.5 ± 14.2	29.8 ± 14.9	38.4 ± 17.3	<.001
Monounsaturated fats, g	26.9 ± 15.9	18.3 ± 12.7	24.9 ± 13.8	33.7 ± 15.2	43.7 ± 18.8	<.001
Polyunsaturated fats, g	18.5 ± 11.6	13.5 ± 9.8	17.8 ± 10.8	22.2 ± 11.9	25.5 ± 13.6	<.001

Abbreviations: BMI, body mass index; WC, waist circumference.

^a^ Values are mean ± SD unless otherwise indicated.

The results from the multinomial logistic regression with crude and multivariable models (adjusted for age, sex, body mass index, energy intake, and total protein) showed that consuming <1 g of dietary creatine per day did not significantly increase the risk of most medical conditions and functional limitations/disabilities (Table 2). However, eating less creatine (<1 g/day) exposed participants to significantly higher risk of having angina pectoris (adjusted OR = 2.62, 95% CI from 1.14 to 6.01, *p* = .023), a liver condition (adjusted OR = 2.59, 95% CI from 1.23 to 5.48, *p* = .013), and pain or discomfort in chest (adjusted OR = 1.71, 95% CI from 1.17 to 2.50, *p* = .005) as compared to participants who ingested ≥1.00 g of creatine daily. In addition, suboptimal creatine consumption was associated with lower risk of having a broken wrist (adjusted OR = 0.52, 95% CI from 0.30 to 0.90, *p* = .02).

**TABLE 2:** Odds ratios (OR) for having medical conditions and functional limitations in elderly individuals consuming <1.0 g of dietary creatine per day

	OR (95% CI)	Adjusted OR (95% CI)
Medical conditions		
Arthritis	1.17 (0.92 – 1.50)	1.22 (0.86 – 1.72)
Gout	0.91 (0.62 – 1.33)	1,01 (0.59 – 1.73)
Congestive heart failure	1.34 (0.86 – 2.10)	0.97 (0.52 – 1.80)
Coronary heart disease	1.11 (0.76 – 1.62)	1.07 (0.63 – 1.81)
Angina pectoris	1.90 (1.05 – 3.45)*	2.62 (1.14 – 6.01)*
Heart attack	1.06 (0.71 – 1.57)	0.99 (0.57 – 1.71)
Stroke	0.89 (0.60 – 1.32)	0.85 (0.48 – 1.50)
Thyroid problem	0.99 (0.73 – 1.33)	1.07 (0.69 – 1.64)
Emphysema	1.04 (0.55 – 1.96)	0.95 (0.40 – 2.28)
Chronic bronchitis	0.88 (0.59 – 1.31)	0.92 (0.52 – 1.60)
COPD	1.04 (0.70 – 1.54)	0.84 (49 – 1.45)
Liver condition	1.63 (0.94 – 2.83)	2.59 (1.23 – 5.48)*
Gallstones	1.21 (0.87 – 1.68)	1.43 (0.90 – 2.29)
Cancer	0.94 (0.71 – 1.25)	1.11 (0.74 – 1.65)
Cardiovascular health		
Pain or discomfort in chest	1.49 (1.13 – 1.97)*	1.71 (1.17 – 2.50)*
Shortness of breath on stairs/inclines	1.04 (0.81 – 1.34)	0.95 (0.67 – 1.34)
Diabetes		
Diabetes	1.00 (0.76 – 1.30)	1.16 (0.79 – 1.70)
Prediabetes	0.92 (0.62 – 1.36)	1.22 (0.70 – 2.11)
Health risk for diabetes	0.91 (0.57 – 1.44)	1.74 (0.91 – 3.30)
Ostepoporosis		
Broken or fractured a hip	0.59 (0.31 – 1.12)	1.01 (0.41 – 2.54)
Broken or fractured a wrist	0.81 (0.55 – 1.18)	0.52 (0.30 – 0.90)*
Broken or fractured spine	1.20 (0.63 – 2.28)	0.61 (0.24 – 1.52)
Ostepoporosis/brittle bones	1.21 (0.21 – 5.77)	1.47 (0.90 – 2.39)
Physical functioning		
Limitations keeping a person from working	1.23 (0.90 – 1.66)	0.99 (0.64 – 1.52)
Limited in amount of work one can do	1.09 (0.85 – 1.40)	1.02 (0.72 – 1.45)
Need special equipment to walk	1.23 (0.93 – 1.63)	1.18 (0.79 – 1.78)
Experience confusion/memory problems	1.28 (0.88 – 1.84)	1.23 (0.74 – 2.06)
Physical, mental, emotional limitations	1.72 (0.81 – 3.68)	1.85 (0.67 – 5.16)
Disability		
Serious difficulty hearing	0.86 (0.64 – 1.15)	0.93 (0.61 – 1.41)
Serious difficulty seeing	1.00 (0.66 – 1.50)	1.37 (076 – 2.49)
Serious difficulty concentrating	0.88 (0.61 – 1.27)	0.66 (0.39 – 1.12)
Serious difficulty walking	1.08 (0.83 – 1.41)	1.04 (0.71 – 1.52)
Difficulty dressing or bathing	0.79 (0.54 – 1.15)	0.78 (0.45 – 1.35)
Difficulty doing errands alone	0.90 (0.64 – 1.27)	0.98 (0.60 – 1.60)
Take medication for depression	1.08 (0.73 – 1.58)	1.43 (0.84 – 2.42)

Note

ORs were adjusted for age, sex, body mass index, energy intake, and total protein. Asterisk (*) indicates statistical significance at *p* < .05.

## 4 DISCUSSION

To our knowledge, this is the first population‐based study that examined dietary intake of creatine and risk of medical conditions in a nationally representative sample of older men and women. We found that U.S. seniors recruited during the NHANES 2017–2018 round consume 0.76 g of dietary creatine per day on average, which is below the amounts required for adults (1.00 g/day). As much as 70% of U.S. elderly consume <1.00 g of creatine per day, with about 1 in 5 individuals (19.8%) consume no creatine at all. Furthermore, elderly who consume less than recommended amounts of creatine were found to have 2.62 times higher risk of angina pectoris, and 2.59 times higher risk of liver conditions, compared with older counterparts who consume ≥1.00 g of creatine per day after controlling for demographic and nutritional variables.

The populational studies exploring creatine intake through a regular diet are surprisingly scarce. Brosnan et al. (2011) were arguably the first who estimated that the regular diet provides daily around 7.9 and 5.0 mmol of creatine (equivalent to 1.18 g and 0.75 g) for U.S. men and women, respectively. This estimation has been based on the NHANES III data on the consumption of different foods between 1988 and 1994 in the 19‐ to 39‐year age group. However, those findings have never been published in a research paper but instead reported as secondary data in a review article (Brosnan et al., 2011). A recent study outlined somewhat lower amounts of daily creatine consumed by adult men (0.67 ± 0.39 g) and women (0.42 ± 0.26 g) aged 20 years or older, using population‐based data from the 2005–2012 NHAHES rounds (Bakian et al., 2020). Using similar methods to the above studies for calculating creatine intake, our findings of average dietary creatine consumption in the elderly (0.76 ± 0.79 g/day, median value 0.57 g) repose in the interval between the results from previous research. Somewhat, different results in creatine consumption could be due to heterogeneity of populations evaluated in different studies, with age‐specific variation in nutritional habits perhaps translates into varying amounts of creatine intake. For instance, older adults consume less meat and meat products than their younger counterparts (Schmid et al., 2017), therefore providing a smaller amount of creatine from foods rich in animal protein. Another factor that could be accounted for a discrepancy between studies' results involves temporal trends in the food habits during the past three decades, mainly illustrated by a reduction in meat consumption in the U.S. population (Neff et al., 2018). Since meat is a major source of creatine, eating less meat will inevitably lead to lower creatine availability, with the elderly perhaps particularly susceptible to this set of circumstances.

Since creatine is a conditionally essential nutrient, no dietary reference intakes and recommended dietary allowances have been established for this nutritional compound (Institute of Medicine, 2006). Still, a torrent of published literature implies that the daily requirement for dietary creatine is at least 1 g for a healthy adult, an amount that should be obtained from a typical omnivorous diet to accompany the portion of creatine synthesized inside the body (Brosnan & Brosnan, 2016). An insufficient intake of dietary creatine (<1.00 g/day) may thus lead to creatine malnutrition that could compromise normal creatine turnover. As an illustration, the individuals who are unable to consume enough creatine from the food sources often demonstrate low‐creatine availability and concomitant health conditions (Bakian et al., 2020; Balestrino & Adriano, 2019). Surprisingly, no populational studies known to the authors explored the prevalence of low‐creatine consumption using an assumed margin of 1.00 g/day. We found here that 7 out of 10 U.S. older individuals consume creatine below this threshold point, making creatine malnutrition widespread in this population. It turns out that 19.8% of participants consumed zero creatine from a regular diet (no‐intake group), while 50.2% of respondents consume some creatine from food yet below the assumed margin of 1.00 g/day (low‐intake group), with average creatine intake across these two categories combined was 0.36 ± 0.31 g/day (median 0.33 g). Whether this considerable shortage of dietary creatine is counterbalanced by an enhanced endogenous synthesis of creatine in the elderly remains currently unknown. Previous studies suggest a compensatory hyper‐production of creatine in young adults eating creatine‐free diets (Watt et al., 2004), yet the net effect of this response on total creatine turnover appears inadequate (Delanghe et al., 1989). This handicap might be accentuated in the elderly who experience reduced tissue creatine content and unfavorable changes in creatine synthesis machinery (Dolan et al., 2019). As such, advocating for appropriate dietary intake of creatine at the populational level might be necessary to support optimal creatine homeostasis in this delicate population.

The researchers from the University of Utah School of Medicine were probably the first who explored the association between creatine intake from a regular diet and health outcomes at the populational level (Bakian et al., 2020). They found a significant negative relationship between creatine consumption and depression in a nationally representative cohort of adults aged ≥20 years, with depression prevalence was significantly higher among participants in the lowest quartile of dietary creatine intake (Q1, 0.15 ± 0.08 g/day) comparing to the participants in the highest quartile of creatine consumption (Q4, 1.01 ± 0.30 g/day). We explored here a link between dietary intake of creatine and relevant medical conditions and physical disabilities among two distinct biologically plausible creatine consumption subpopulations rather than using distribution‐based quartiles. We found that the suboptimal creatine intake (<1.00 g/day) increased the risk for pain or chest discomfort (adjusted OR 1.71) and angina pectoris (adjusted OR 2.62). This means that the older participants who regularly consume creatine below the threshold value have up to 2.62 times higher risk to have ischemic heart disease‐related traits compared with peers whose dietary intake of creatine is ≥1.00 g/day. Given creatine's essential role in protecting the cardiac cells sarcolemma against ischemic damage (Guzun et al., 2011), an inadequate provision of creatine through a regular diet may compromise normal heart function and perhaps contribute to myocardial ischemic conditions in the elderly. In contrast, adding creatine to a therapeutic cocktail in acute myocardial ischemic conditions improves the hemodynamic response and clinical conditions (Landoni et al., 2016). Having this in mind, creatine suboptimal intake should be perhaps recognized as another nutritional factor that can negatively affect heart health in the elderly, and a condition that requires appropriate care. Since cardiovascular diseases (CVDs) remain a major cause of health loss for all regions of the world (Roth et al., 2017), addressing creatine malnutrition might be considered among innovative public measures focused on reducing the overall burden of CVDs, either via fostering diets rich in creatine‐containing foods, creatine supplementation, and/or food fortification with creatine (Ostojic, 2020). Besides, low‐creatine intake significantly increased the risk of liver conditions (adjusted OR 2.59) when adjusted for age, sex, body mass index, energy intake, and total protein in the U.S. elderly. Since the liver is an energy‐demanding organ that plays a key role in creatine synthesis (Wyss & Kaddurah‐Daouk, 2000), inadequate creatine provision from food sources may perturb normal creatine turnover and induce a hepatic malfunction. In line with this premise, low tissue creatine levels are reported in a wide range of hepatic disorders (Dagnelie & Leij‐Halfwerk, 2010; Scheau et al., 2012), while creatine consumption is known to reduce the homocysteine production in the liver and diminish fat accumulation resulting in beneficial effects in fatty liver and nonalcoholic liver diseases (Barcelos et al., 2016; Deminice et al., 2016). Our results thus support the findings from creatine interventional trials and perhaps support the idea that the elderly may benefit from dietary creatine intake in the prevention and/or management of liver conditions.

Several limitations must be considered when study findings are interpreted. We employed here a general recommendation for dietary requirements of creatine (1.00 g/day) for the adult population since no age‐specific criterion on dietary needs for creatine is currently available. This may, however, overestimate the number of participants with creatine malnutrition since aging likely reduces the total creatine pool and creatine demand. Future trials also have to determine the rate of de novo creatine synthesis across different demographic cohorts and nutritional profiles to control for this component in computing balance (and needs) between endogenous and exogenous creatine load. Another possible limitation includes the premise that dietary data and medical information collected through the self‐ and proxy‐reported NHANES questionnaires provide a valid tool of participants' nutrition and health. Although direct measures are highly warranted to provide more detailed information, NHANES tools appear to produce reliable estimates at the populational level while being minimally burdensome (Ahluwalia et al., 2016).

## 5 CONCLUSION

A majority of U.S. elderly consume dietary creatine below the amounts recommended for adults, making creatine malnutrition rather widespread in this sensitive population. The considerable shortage of dietary creatine is associated with an increased risk of heart and liver conditions, which calls for public nutrition measures that foster diets rich in creatine‐containing foods, and additional research to investigate the role of creatine in age‐related diseases.

## CONFLICT OF INTEREST

S.M.O. serves as a member of the Scientific Advisory Board on creatine in health and medicine (AlzChem LLC). S.M.O. owns patent ‘Sports Supplements Based on Liquid Creatine’ at European Patent Office (WO2019150323 A1), and active patent application ‘Synergistic Creatine’ at UK Intellectual Property Office (GB2012773.4). S.M.O. has served as a speaker at Abbott Nutrition, a consultant of Allied Beverages Adriatic and IMLEK, and an advisory board member for the University of Novi Sad School of Medicine, and has received research funding related to creatine from the Serbian Ministry of Education, Science, and Technological Development, Provincial Secretariat for Higher Education and Scientific Research, AlzChem GmbH, KW Pfannenschmidt GmbH, and ThermoLife International LLC. S.M.O. is an employee of the University of Novi Sad and does not own stocks and shares in any organization. D.K. and V.S. declare no conflict of interest.

## AUTHOR CONTRIBUTIONS

**Sergej M. Ostojic:** Conceptualization (equal); Data curation (equal); Formal analysis (equal); Funding acquisition (equal); Investigation (equal); Methodology (equal); Project administration (equal); Resources (equal); Software (equal); Supervision (equal); Validation (equal); Visualization (equal); Writing‐original draft (equal); Writing‐review & editing (equal). **Darinka Korovljev:** Data curation (equal); Formal analysis (equal); Investigation (equal); Methodology (equal); Resources (equal); Validation (equal); Writing‐original draft (equal). **Valdemar Stajer:** Data curation (equal); Formal analysis (equal); Investigation (equal); Methodology (equal); Resources (equal); Software (equal); Writing‐original draft (equal).

## ETHICAL APPROVAL

The study conforms to the Declaration of Helsinki, US, and European Medicines Agency Guidelines for human subjects. The study's protocols and procedures were ethically reviewed and approved by the U.S. NCHS Research Ethics Review Board (#2018‐01 and #2011‐17), and informed consent was obtained from all respondents.
